# Anti-Hyperlipidemia and Gut Microbiota Community Regulation Effects of Selenium-Rich *Cordyceps militaris* Polysaccharides on the High-Fat Diet-Fed Mice Model

**DOI:** 10.3390/foods10102252

**Published:** 2021-09-23

**Authors:** Minglei Yu, Jin Yue, Nan Hui, Yuee Zhi, Kashif Hayat, Xijia Yang, Dan Zhang, Shaohua Chu, Pei Zhou

**Affiliations:** 1 School of Agriculture and Biology, Shanghai Jiao Tong University, Shanghai 200240, China; fish_sunshine@sjtu.edu.cn (M.Y.); nan.hui@sjtu.edu.cn (N.H.); yueezhi@sjtu.edu.cn (Y.Z.); khayat97@sjtu.edu.cn (K.H.); eileenyang33@163.com (X.Y.); zhdsjtu@sjtu.edu.cn (D.Z.); chushyt@sjtu.edu.cn (S.C.); 2 Key Laboratory of Urban Agriculture, Ministry of Agriculture and Rural Affairs of the PRC, Shanghai 200240, China; 3 Bor S. Luh Food Safety Research Center, Shanghai Jiao Tong University, Shanghai 200240, China; jinyue@sjtu.edu.cn

**Keywords:** *Cordyceps militaris*, selenium-rich crude polysaccharides, high-fat diet (HFD), dyslipidemia, hypertriglyceridemia, gut microbiota

## Abstract

Supplementation of polysaccharides is a promising gut microbiota-targeted therapeutic method for obesity and metabolic diseases. Biological activities of *Cordyceps militaris* polysaccharides have been well reported, but the effect of selenium (Se)-rich *C. militaris* polysaccharides (SeCMP) on obesity and associated metabolic disorder and gut microbiota composition has been rarely studied. This study aimed to investigate the anti-obesity and gut microbiota modulatory effect of crude polysaccharides separated from Se-rich *C. militaris* on a high-fat diet (HFD)-fed C57BL/6J mice model. Mice were treated with a normal diet (CHOW), HFD alone, HFD plus *C. militaris* polysaccharides (CMP), or low/medium/high dosage of SeCMP for 8 weeks. Body weight, fat content, serum lipid, appetite hormone, lipid gene expression, inflammation cytokines, thermogenic protein, short-chain fatty acids (SCFAs), and gut microbiota structure of the mice were determined. Compared with HFD-fed mice, the serum triglyceride and low-density lipoprotein cholesterol (LDL-C) in the SeCMP-200 group were decreased by 51.5% and 44.1%, respectively. Furthermore, serum lipopolysaccharide-binding proteins (LBP), adiponectin level, and pro-inflammation gene expression in the colon and subcutaneous fat were inhibited, whereas anti-inflammation gene expression was improved, reflecting SeCMP-200 might mitigate obese-induced inflammation. Meanwhile, SeCMP-200 promoted satiety and thermogenesis of obese mice. It also significantly decreased gut bacteria, such as *Dorea*, *Lactobacillus*, *Clostridium*, *Ruminococcus,* that negatively correlated with obesity traits and increased mucosal beneficial bacteria *Akkermansia*. There was no significant difference between CMP and SeCMP-100 groups. Our results revealed a high dose of SeCMP could prevent HFD-induced dyslipidemia and gut microbiota dysbiosis and was potential to be used as functional foods.

## 1. Introduction

Obesity and associated metabolic disorders have become international public health issues that increase a series of severe health problems, such as type-2 diabetes, fatty liver diseases, cardiovascular diseases, and cancers [1]. Organic compounds in low molecular weight predominate the medications list of obesity. Nevertheless, their efficacy to different individuals is varied, and they may cause severe side effects. In addition, they bring about many environmental issues due to large amounts of organic solvents used for production [2,3,4]. It is very challenging to develop effective, safe, and inexpensive nutraceuticals as adjuvant medical therapy to the present drugs. Bio-macromolecules from plants are some promising natural candidates.

*Cordyceps militaris* is a cultured edible mushroom popularized in Asian countries [5]. The biological activity of the polysaccharides in *C. militaris* has given rise to great attention in recent years. The polysaccharides comprise three main monosaccharides, mannose, glucose, and galactose with the backbone characterized by (1→4)-*β*-D-Glc*p*, (1→6)-linked-*α*-D-Glu*p*, (1→3)-*β*-D-Glc*p,* and (1→2)-*α*-D-Man*p* [6,7,8,9]. These complex structures are correlated with immunomodulatory effects attributed to *α*- and *β*-D-glucosidic linkages [8]. They can not only stimulate macrophages to secrete nitric oxide (NO), tumor necrosis factor-alpha (TNF-*α*), interleukin-10 (IL-10), and interleukin-6 (IL-6) and increasing M1-polarized macrophages [7,9] but also promote splenic lymphocytes proliferation for immunity’s sake. Furthermore, *C. militaris* polysaccharides exhibited strong inhibition of *α*-glucosidase activity, suggesting it may reduce postprandial elevated blood glucose and prevent diabetes [10]. *C. militaris* extract fermented with gut microbiota in vitro affected the gut microbiota composition and metabolites significantly, especially decreasing the ratio of Firmicutes to Bacteroidetes, reducing pH, and improving propionic acid production [11].

Mushrooms accumulate many microelements from the soil or growth medium, including selenium (Se), which makes them a valuable dietary source of Se [12]. Se is well known for its antioxidant activity effect in redox homeostasis, intracellular signaling regulation, and induction of apoptosis in transformed cells [13]. Recently, the function of Se on obesity and the gut has received growing attention. Se-containing complexes showed a strong effect on reducing the serum and liver lipids and atherogenic index by regulating leptin, inflammation response, and enhancing the activities of antioxidant enzymes of obese mice [14,15]. Furthermore, they can alleviate organ damage caused by obesity attributed to the inflammatory response reduction effect [16]. Experimental studies have shown that gut microflora is able to metabolize inorganic and organic seleno-compounds to improve the bioavailability of seleno-compounds for the purpose of the host’s selenium status maintenance [17]. Se dietary supplements improved gut barrier and immune response through regulating host gut microbiota composition [18,19]. However, this is not always the case. Gao et al. found Se-rich and Se-deficient black tea act similarly in fat accumulation and gut microbiota dysbiosis prevention [20].

This study aimed to determine the effect of polysaccharides in Se-rich *C. militaris* (SeCMP) to prevent obesity and regulate the gut microbiota. An HFD-fed mice model was adopted in this work. The advent of high-throughput sequencing combined with bioinformatics substantially boosted our ability to identify bacterial biomarkers that can predict an individual’s response to the treatments.

## 2. Materials and Methods

### 2.1. Materials and Reagents

*C. militaris* strain was isolated from wild fruit bodies by the Institution of Microbiology, Chinese Academy of Sciences (Beijing, China). Se-rich *C. militaris* and Se-deficient *C. militaris* fruit bodies were purchased from Nanguang Biotech Develop Ltd., (Shanghai, China) and identified by Bin Li (Associate research fellow, Chinese Academy of Sciences, Beijing, China). Se content in the Se-deficient and Se-rich *C. militaris* fruit bodies were 0.1 ± 0.0 and 3.9 ± 0.6 mg/kg, respectively. Chemical reagents were analytical grade and purchased from Sinopharm Chemical Reagent Co. Ltd. (Shanghai, China) or Lingfeng Chemical Reagent Co. Ltd. (Shanghai, China).

### 2.2. Isolation of Crude Polysaccharides

Se-rich *C. militaris* and Se-deficient *C. militaris* fruit bodies were dried and ground into powder (80 mesh). A mixture of the dried powder and distilled water (1:10, *w*/*v*) was ultrasonically concussed for 30 min at 50 °C and boiled at 121 °C for 30 min. The solution was centrifuged at 4000 rpm for 15 min at room temperature using the Bioridge TD5M-WS centrifuge (Lu Xiangyi Centrifuge Instrument Co. Ltd., Shanghai, China). The supernatants were concentrated to 1/4 volume at 65 °C using the EYELA SB1100 vacuum concentrator (Tokyo, Japan). Absolute ethyl alcohol (1.5 L) was added to the 500 mL concentration and stored at 4 °C overnight. The precipitates, namely CMP and SeCMP, were collected and lyophilized into powder using the Virtis BenchTop Pro freeze dryer (Virtis, Pennsylvania, USA). Crude polysaccharide was determined using the phenol–sulfuric acid method [21]. Five milliliters of sulfuric acid was quickly added into tubes with 1 mL of 5% phenol and 1 mL of sample solution. The obtained solutions were heated in a water bath at 100 °C for 20 min, then cooled to ambient temperature, and finally, its absorbance at 490 nm was measured. Crude proteins were determined by the Kjeldah method [22]. Crude fat was extracted by petroleum ether with the Soxhlet extractor for 6 h and followed by solvent evaporation [23]. Amino acids were determined by an amino acid analyzer (L-8900, Hitachi High-Tech, Tokyo, Japan) after samples were totally hydrolyzed by hydrochloric acid [24]. Total phenol content was determined using the Folin–Ciocalteu reagent. The sample solution was added to the mixture of Folin–Ciocalteu reagent and 15% Na_2_CO_3_. It was kept at 40 °C in the dark for 60 min before the absorbance was monitored at 760 nm [25,26]. The Se content of the samples was determined by an atomic absorption spectrometer (AAS, AA800, PerkinElmer, Waltham, MA, USA) after the sample solutions were totally digested in a microwave oven using nitric acid and hydrogen peroxide [27]. Total diet fiber was identified using an enzymatic gravimetric method (AOAC Methods 2011.25). Moisture and ash content were determined using the gravimetric method. The mass of carbohydrate and energy were calculated from Formulas (1) and (2), respectively.

*m*_Carbohydrate_ (g) = *m*_Total sample_ (g) − *m*_protein_ (g) − *m*_Moisture_ (g) − *m*_Ash_ (g) − *m*_Diet fiber_ (g) (1)

*E* (kcal) = 4 (kcal/g) × *m*_Carbohydrate_ (g) + 9 (kcal/g) × *m*_Fat_ (g) + 4 (kcal/g) × *m*_Protein_ (g) (2)

where *m* is mass and *E* is energy of each polysaccharides extract.

### 2.3. Animal Experiments

The animal study was approved by the Institutional Animal Care and Use Committee of Shanghai Jiao Tong University (IACUC; No. 2018089). Forty-eight four-week-old male specific-pathogen-free C57BL/6J mice (purchased from the Beijing Vital River Laboratory Animal Technology Co. Ltd., Beijing, China) were raised, 4 mice/cage in a controlled environment (temperature at 26 ± 1 °C, 12-h dark-light cycle). After 1-week acclimation, mice were divided into 6 groups (8 mice/group) with free access to normal or HFD diets. Sterile water, SeCMP, and CMP were administered by intragastric gavage. Mice were fed with (1) normal diet + sterile water (CHOW group); (2) HFD + sterile water (HFD group); (3) HFD + 100 mg/kg bw/day CMP (CMP group); (4) HFD + 50 mg/kg bw/day SeCMP (SeCMP-50 group); (5) HFD + 100 mg/kg/day SeCMP (SeCMP-100 group); (6) HFD + 200 mg/kg/day SeCMP (SeCMP-200 group). The normal diet with 5.5% energy from fat and HFD with 60% energy from fat were purchased from Xinhui Animal Stall Food Co. Ltd. (Shanghai, China). The mice were weighed once a week throughout the experiment period. Food and water consumption of each cage was monitored twice a week and converted into daily intake per mouse. The lipid content in stools was assessed by gravimetric analysis procedures [28]. At the end of the animal study, fasting animals were anesthetized with isoflurane, and then the blood was collected by cardiac puncture. After being rested in ambient temperature for more than 30 min, the blood was centrifuged by a CT14RD high-speed refrigerated centrifuge (Tianmei Biochemical Instrument and Equipment Engineering Co. Ltd., Shanghai, China) at 2000× *g* under 4 °C for 20 min. Supernatant serum was collected and placed in EP tubes and then stored at −80 °C for further analysis. Tissues were carefully removed and weighed, including liver, heart, spleen, lung, kidney, pancreas, brown adipose fat, and white adipose fat (white adipose fat including subcutaneous, epididymal, and perirenal fat). The tissues for staining were preserved in formalin solution (Sangon biotech, Shanghai, China) for the following study. The rest of the tissues and stool samples were snap-frozen in liquid nitrogen after separation from the body and stored at −80 °C.

### 2.4. Serum Biochemical Analyses

Serum lipid indexes, including total cholesterol (TC) (Product ID: A111-1-1), triglyceride (TG) (Product ID: A110-1-1), high-density lipoprotein-cholesterol (HDL-C) (Product ID: A112-1-1), and low-density lipoprotein-cholesterol (LDL-C) (Product ID: A113-1-1), as well as liver and renal function indexes, such as alanine aminotransferase (ALT) (Product ID: C009-2-1), aspartate aminotransferase (AST) (Product ID: C010-2-1), uric acid (UA) (Product ID: C012-2-1), creatinine (CRE) (Product ID: C011-2-1), albumin (ALB) (Product ID: A028-2-1), and total bilirubin (T-BIL) (Product ID: C019-1-1) were quantified using a commercial kit purchased from Nanjing Jiancheng Bioengineering Institute Co. Ltd. (Nangjing, China). Serum leptin (Product ID: PL696, Biyotime biotechnology, Shanghai, China) and adiponectin (Product ID: PA002, Biyotime biotechnology, Shanghai, China) and LBP (Product ID: ab213876 -1 × 96 test, Abcam, Cambridge, UK) were measured with ELISA kits.

### 2.5. Oil Red O Staining

The fixed liver tissues were stained with Oil Red O (Servicebio, Wuhan, China) for fat coloring. The slide was rinsed with 75% of ethyl alcohol for histo-differentiation and then washed with distilled water. The nuclei of the stained tissues were dyed blue with hematoxylin. Sections were examined with the NIKON ECLIPSE TI-SR inverted fluorescence microscope (NIKON, Tokyo, Japan), and scanned sections were captured using the NIKON DS-U3 microscope camera controller (NIKON, Tokyo, Japan). Mean adipose area was quantified with 3 tissue sections for each animal using Image J (v1.8.0).

### 2.6. Histopathological Staining

The previous fixed epididymal white adipose tissues (eWATs) were stained with hematoxylin and eosin (H&E). The slide scanned machine and mean adipocyte size quantified software were the same as those used in Section 2.5.

### 2.7. Immunohistochemistry (IHC) Staining

The white adipose tissues (WATs) and brown adipose tissues (BATs) sections were hybridized with rabbit anti-mouse uncoupling protein 1 antibody (UCP1, Abcam, UK) before being exposed to 3,3-diaminobenzidine (DAB). Cell nuclei were counterstained with hematoxylin. The scanning instrument and protein expression quantification method were the same as that used in Section 2.5.

### 2.8. RNA Extraction and q-PCR Analysis

TRIzol reagent (Invitrogen, Carlsbad, CA, USA) was utilized to extract total RNA from liver, subcutaneous fat, and colon. The extracts were then used to synthesize cDNA with a PrimeScript RT reagent kit with gDNA Eraser (Takara Biotechnology, Dalian, China). Q-PCR was performed using TB Green^®^ Premix Ex Taq™ II (Tli RNaseH Plus) (Takara Biotechnology, Dalian, China) on the Applied Biosystems StepOne Real-Time PCR instrument (Life Technologies, Carlsbad, CA, USA). Relative amounts of target genes were normalized to the housekeeping gene GAPDH and calculated based on the 2^−ΔΔCt^ method. Genes and primer sequences are listed in Table S5.

### 2.9. Quantification of Short-Chain Fatty Acids (SCFAs) in the Stool by GC-MS

Stools were dispersed in deionized water. The supernatants were acidified with 50% H_2_SO_4_ and extracted with ethyl ether. The extracts were subsequently subjected to SCFAs for determination. Acetate, propionate, butyrate, isobutyric acid, valeric acid, and isovaleric acid were identified by GC-MS (Agilent 7890B-7000D) with a 30 m × 0.25 mm × 0.25 μm free fatty acid phase (FFAP) capillary column (Agilent Technologies, Santa Clara, CA, USA). The injector temperature was 250 °C. The oven temperature was set at 100 °C which was maintained for 1 min, increased to 145 °C at 5 °C/min, and finally raised to 240 °C at 15 °C/min, and maintained at 240 °C for 8 min. Ion source temperature: 230 °C; ionization mode: EI+, 70 eV; scanning mode: selected ion monitor (SIM). SCFAs contents were calculated according to the standard curve of the above 6 SCFAs.

### 2.10. Cecal Microbiota DNA Extraction

Total bacterial genomic DNA was extracted using the Mag-Bind^®^ DNA Kit (QIAGEN, Dusseldorf, Germany). The V3-V4 regions of 16S rRNA were amplified using composite primers containing the forward primer 338F (5′-ACTCCTACGGGAGGCAGCA-3′) and the reverse primer 806R (5′-GGACTACHVGGGTWTCTAAT-3′). The PCR amplicons were purified using Agencourt AMPure Beads (Beckman Coulter, Indianapolis, IN, USA), then quantified with the PicoGreen dsDNA Assay Kit (Invitrogen, Garlsbad, CA, USA). The amplicons were pooled into equal amounts and paired-end 2 × 300 bp sequencing was performed on an Illumina MiSeq platform with an MiSeq Reagent Kit V3 at the Shanghai Personal Biotechnology Co. Ltd. (Shanghai, China). The sequence data were submitted to the NCBI sequence read archive (SRA) (succession number: SRR12927607-SRR12927629, SRR13644816-SRR13644827).

### 2.11. Bioinformatics and Statistics

Sequence data analyses were performed with QIIME 2 software (2019.4) with slight modification according to the official tutorials. Briefly, raw sequence data were demultiplexed, followed by primers cutting. Sequences were then qualified for high-quality reads through filtering, denoising, merging, and the chimera removal process using the DADA2 plugin. Non-singleton amplicon sequence variants (ASVs) were aligned and used to construct a phylogeny. To avoid sampling depth bias, the abundance of ASVs were randomly rarefied from each sample to reach a uniform depth (at 95% of the smallest sample amounts) (Qiime feature-table rarefy). Alpha-diversity metrics Chao1, Simpson, and Pielou’s evenness indexes were calculated using QIIME2. Beta diversity analysis was performed to investigate the structural variation of microbial communities across samples using Bray–Curtis distance-based nonmetric multidimensional scaling (NMDS) in QIIME2. The significance of differentiation of microbiota structure among groups was assessed by PERMANOVA (Permutational multivariate analysis of variance) and ANOSIM (Analysis of similarities) using R software. Taxonomy was assigned to ASVs using the classify-sklearn naìve Bayes taxonomy classifier in the feature-classifier plugin against the Greengenes 13_8 [29] 99% OTUs reference sequences. Taxa abundances at the phylum, class, order, family, genus, and species levels were statistically compared between groups by multivariate statistical analysis. LEfSe (Linear discriminant analysis effect size) was performed to detect differentially abundant taxa across groups using Python LEfSe package and R software.

Data were assessed using one-way analysis of variance (ANOVA) followed by Tukey’s post hoc test or the non-parametric Kruskal–Wallis test with Dunnett’s T3 multiple comparison test by GraphPad Prism 8.0 (GraphPad Software Inc., San Diego, CA, USA). The chemical content in CMP and SeCMP was compared using a two-tailed independent *t*-test. Data were presented as mean ± standard error (SE) of triplicate analyses. Correlation coefficients between bacterial species and obesity traits were determined using Pearson’s correlation analysis with Origin 2021. *p* < 0.05 was considered to be statistically significant.

## 3. Results

### 3.1. Chemical Compositions in SeCMP and CMP

The descriptive statistics of the experimental data are presented in Table 1. The yield of CMP and SeCMP was 14.3 ± 0.7% and 15.2 ± 0.9%, respectively. The most abundant components were crude polysaccharides and protein, which constituted 47.4, 35.1% in CMP, and 56.75%, 31.1% in SeCMP. There was 5.1 mg/kg of Se in SeCMP, 50 times higher than that in CMP (*p* < 0.001).

### 3.2. Body Weight, Adipocyte Size, and Liver Steatosis

**Figure 1:**
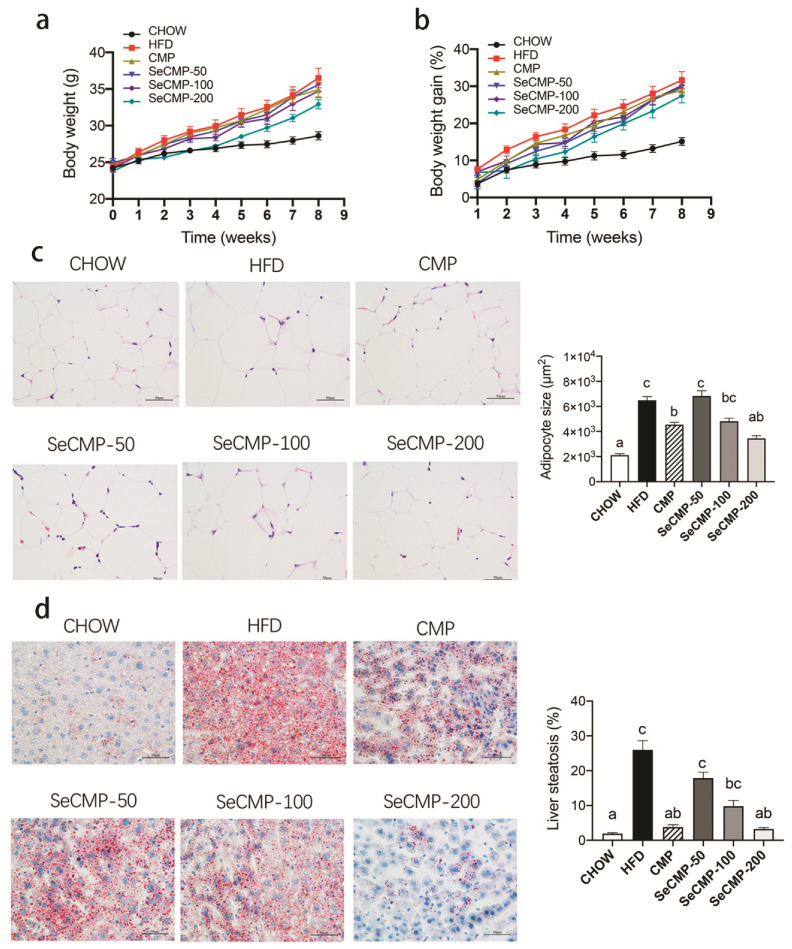
The effect of selenium-rich *C. militaris* crude polysaccharides (SeCMP) and selenium-deficient *C. militaris* crude polysaccharides (CMP) on body weight and alleviated lipid accumulation in high-fat diet-induced obese C57BL/6J mice. (**a**) The body weight, (**b**) body weight gain throughout the experiment, (**c**) mean adipocyte size, and (**d**) liver steatosis ratio stained with oil-red O dye. Scale bars: 50 μm. Data are expressed as mean ± SE (n = 8 mice/group). Superscript characters indicate significant variation among treatments. CHOW: normal diet + sterile water; HFD: high-fat diet + sterile water; CMP: high-fat diet +100 mg/kg CMP; SeCMP-50: high-fat diet +50 mg/kg SeCMP; SeCMP-100: high-fat diet +100 mg/kg SeCMP; SeCMP-200: high-fat diet +200 mg/kg SeCMP.

Daily administration of SeCMP or CMP did not affect food intake, water intake, nor stool fat excretion (Figure S1), indicating SeCMP and CMP modulating the accumulation of tissue fat might be driven by the physiological changes instead of affecting lipid absorption and excretion [30]. Compared with the control CHOW group, HFD significantly increased body weight (*p* < 0.0001) (Figure 1a,b) of mice. The body weight in all the other groups had no significant difference from that of the HFD group (Figure 1a,b).

Histological staining result showed HFD supplementation elevated the adipocytes size (*p* < 0.0001) (Figure 1c) and lipid accumulation in liver (*p* < 0.0001) (Figure 1d). Compared with the HFD group, CMP and SeCMP-200 treatments could significantly decrease both the adipocyte size and liver steatosis, especially the SeCMP-200 treatment, which could reduce them to the level of the CHOW treatment. It indicated that high dose SeCMP intake can ameliorate HFD-induced lipid accretion in adipocyte and liver.

As shown in Table 2, HFD significantly increased the weight gain of WATs (white adipose tissues), including perirenal fat (*p* < 0.0001), epididymal fat (*p* < 0.0001), subcutaneous fat (*p* < 0.0001), and BAT (brown adipose tissue) (*p* < 0.0001) of mice. Among all the groups treated with CMP or different levels of SeCMP, there were no significant differences in WATs or BAT weight. Only the SeCMP-200 group was observed to have significantly reduced fat accretion in lipid depots (*p* < 0.05), except BAT. Compared with the CHOW group, the liver weight of HFD significantly decreased (*p* < 0.0001). It indicated that HFD might cause changes in the liver, whereas the underlying mechanism needs further exploration. In addition, organs, such as the pancreas, heart, spleen, lung, and kidney, remained intact after each intervention (Table S1).

### 3.3. Effect of SeCMP and CMP on Serum Lipid Level and the Metabolism

**Figure 2:**
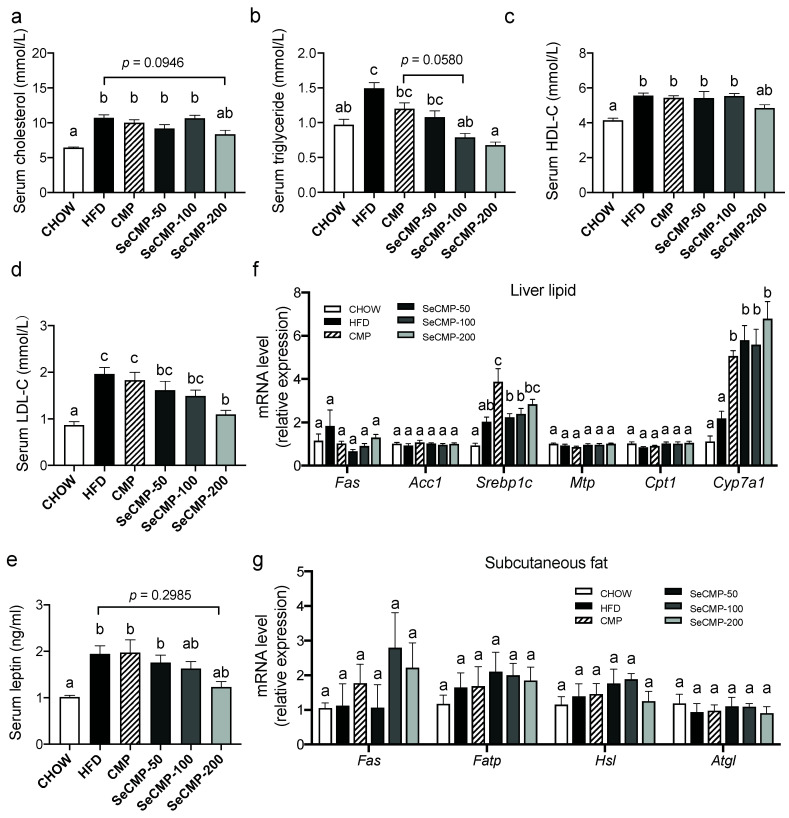
The modulation effect of selenium-rich *C. militaris* crude polysaccharides (SeCMP) and selenium-deficient *C. militaris* crude polysaccharides (CMP) on dyslipidemia in obesity mice. Serum cholesterol, triglycerides, HDL-C, LDL-C, and leptin are shown from (**a**–**e**). Q-PCR analysis was performed to quantify lipid absorption, distribution, and metabolism gene expression in (**f**) liver and (**g**) subcutaneous adipose tissue. The gene expression involved lipogenesis (fatty acid synthase, Fas; acetyl-CoA carboxylase, Acc1; sterol regulatory-element binding protein 1c, Srebp1c), lipid transport (microsomal triglyceride transfer protein, Mtp; fatty acid transport protein, Fatp), lipolysis (hormone-sensitive lipase, Hsl; adipose triglyceride lipase, Atgl; cholesterol 7α-hydroxylase, Cyp7a1), or β-oxidation (carnitine palmitoyltransferase 1, Cpt1). Data represent mean ± SE (n = 8 mice/group). Superscript characters indicate significant variation among treatments in panel (**a**–**e**). Superscript characters in panel (**f**,**g**) indicate significant variation among treatments of each gene. CHOW: normal diet + sterile water; HFD: high-fat diet + sterile water; CMP: high-fat diet +100 mg/kg CMP; SeCMP-50: high-fat diet +50 mg/kg SeCMP; SeCMP-100: high-fat diet +100 mg/kg SeCMP; SeCMP-200: high-fat diet +200 mg/kg SeCMP.

The serum triglyceride (TG) (Figure 2b) in the HFD group were significantly higher (1.40 ± 0.09 mmol/L) than that in the CHOW group (1.05 ± 0.08 mmol/L) (*p* < 0.0001). With the increasing amount of SeCMP feeding, TG decreased. The TG in the SeCMP-100 (0.79 mmol/L) and SeCMP-200 (0.68 mmol/L) groups were even lower than that in the CHOW group. The serum LDL-C (Figure 2d) in all the treated groups appeared to have the same variation pattern as TG, which indicated SeCMP could be a hyperlipidemia scavenger. However, all the CMP and SeCMP treatments could not significantly reduce the serum cholesterol (Figure 2a) or HDL-C (Figure 2c). In addition, serum parameters AST, CRE, and UA levels were improved by SeCMP-200 administration compared with the CHOW group (Table S2), indicating SeCMP-200 might benefit hepatic and kidney function. SeCMP-200 could control hyperlipidemia of HFD-fed obese C57BL/6J mice.

The level of the adipocyte-secreted hormone leptin was upregulated by HFD feeding (Figure 2e), suggesting HFD caused leptin resistance. With the increasing supplementation of SeCMP, the leptin content decreased gradually, and SeCMP-200 supplementing could reduce the serum leptin (*p* = 0.2958) level to a certain extent in comparison with the HFD group. It demonstrated that supplementation of SeCMP might affect appetite.

In order to elucidate the potential effect of SeCMP and CMP on lipid absorption and metabolism, we measured serum cytokine (Figure 2e) and gene expression, including lipogenesis, transport, lipolysis, β-oxidation in hepatic (Figure 2f) and adipose tissues (Figure 2g). Most gene expression did not fluctuate significantly among groups. Among the lipid-relating genes, Cyp7a1, which performs as a rate-limiting enzyme in the transformation of cholesterol into bile acid (BA), was sharply enhanced in all the polysaccharides treated groups (*p* < 0.05). It implied that SeCMP and CMP might prevent obesity by accelerating the cholesterol metabolism pathway.

### 3.4. SeCMP Prevented Inflammation in HFD-Induced Obese C57BL/6J Mice Model

**Figure 3:**
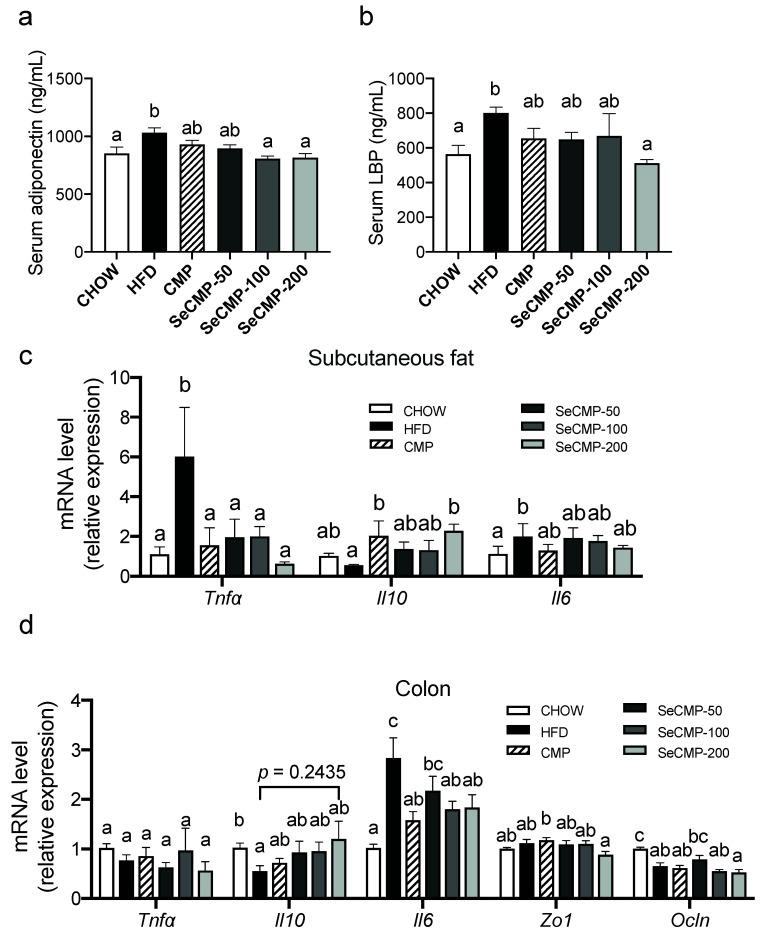
Administration of selenium-rich *C. militaris* crude polysaccharides (SeCMP) alleviated inflammation in C57BL/6J obese mice. (**a**) Serum lipopolysaccharide-binding protein (LBP); (**b**) adiponectin; (**c**) subcutaneous fat inflammation cytokine genes, including tumor necrosis factor-alpha (Tnf-α), interleukin-10 (Il10), and interleukin-6 (Il6); (**d**) Colon inflammation cytokine gene expression, including Tnf-α, Il10, and Il6 and tight junction genes (Zonula occludens 1, Zo1 and Occludin, Ocln). Data represent mean ± SE (n = 6 mice/group). Superscript characters indicate significant variation among treatments in panel (**a**,**b**). In panel (**c**,**d**), superscript characters indicate significant variation among treatments of each gene. CHOW: normal diet + sterile water; HFD: high-fat diet + sterile water; CMP: high-fat diet + 100 mg/kg CMP; SeCMP-50: high-fat diet +50 mg/kg SeCMP; SeCMP-100: high-fat diet +100 mg/kg SeCMP; SeCMP-200: high-fat diet +200 mg/kg SeCMP.

Circulatory and tissues inflammation cytokines were measured to investigate the chronic low-grade inflammation inhibition effect of SeCMP and CMP. Serum adiponectin levels (Figure 3a) and LPS-binding protein (LBP) (Figure 3b) were upregulated by HFD notably (*p* < 0.05) in comparison with the CHOW group, suggesting a higher level of inflammation induced by HFD intake. SeCMP-200 administration reduced LBP level (*p* < 0.01) of HFD-fed mice, and both SeCMP-100 and SeCMP-200 reduced adiponectin level (*p* < 0.05), which implies a high dose of SeCMP supplementation has anti-inflammatory capacity in obese mice. In addition, SeCMP suppressed subcutaneous fat pro-inflammatory cytokine Tnf-α (*p* < 0.05) and upregulated anti-inflammatory cytokine Il10 (*p* < 0.05) expression (Figure 3c). In the colon, SeCMP-200 significantly downregulated inflammatory cytokine Il6 (*p* < 0.05) and increased the anti-inflammatory cytokine Il10 (*p* = 0.2435) of HFD mice (Figure 3d). To sum up, SeCMP-200 regulated inflammatory levels by inducing anti-inflammatory cytokine and suppressing pro-inflammatory cytokine simultaneously in the HFD-induced obese mice.

### 3.5. SeCMP Affected BAT UCP1 Protein Expression in HFD-Induced Obese Mice

**Figure 4:**
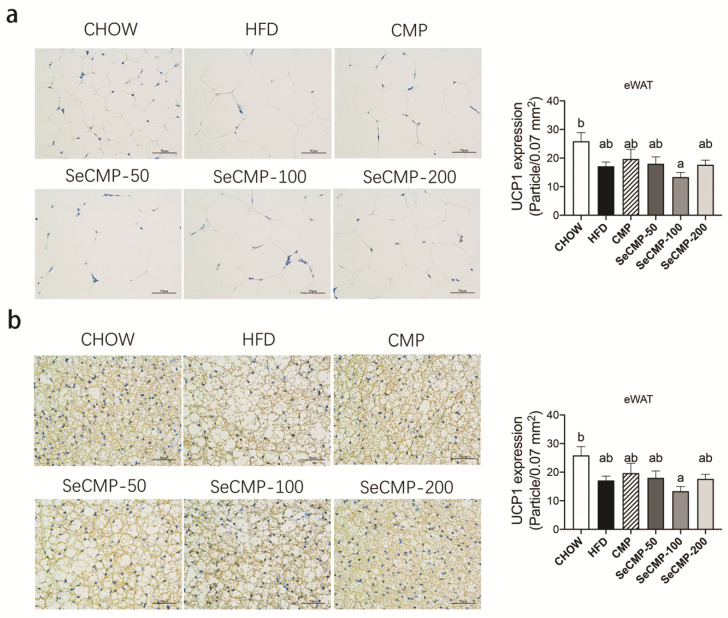
Administration of selenium-rich *C. militaris* crude polysaccharides (SeCMP) improved thermogenic of high-fat diet (HFD)-induced obese mice. (**a**) UCP1 protein expression density in eWAT; (**b**) UCP1 protein expression density in BAT. Scale bars, 50 µm. Data represent mean ± SE (n = 6–8 mice/group). Superscript characters in panel (**a**,**b**) indicate significant variation among treatments. CHOW: normal diet + sterile water; HFD: high-fat diet + sterile water; CMP: high-fat diet +100 mg/kg selenium-deficient *C. militaris* crude polysaccharides; SeCMP-50: high-fat diet +50 mg/kg SeCMP; SeCMP-100: high-fat diet +100 mg/kg SeCMP; SeCMP-200: high-fat diet +200 mg/kg SeCMP.

SeCMP administration groups improved the BAT morphology densification and UCP1 protein expression in BAT in a dose-dependent manner (Figure 4b). Among all the treatment groups, only the SeCMP-200 group showed the most significant increase in BAT UCP1 expression compared with the HFD group (*p* < 0.0001). There was no statistically significant difference between CMP and SeCMP-100 treatments. Meanwhile, eididymal WATs were not significantly affected by either SeCMP or CMP treatment (Figure 4a). It suggested SeCMP-200 increased the BAT thermogenesis in obese mice, which might relate to lipid accumulation and dyslipidemia ameliorating.

### 3.6. SeCMP and CMP Did Not Affect SCFAs in HFD-Induced Obese Mice

SCFAs and branched-chain fatty acids (BCFAs) were microbial metabolites of carbohydrate and protein from dietary. SeCMP or CMP feeding neither changed the caecal production of SCFAs (acetic acid, propionic acid, butyric acid, and valeric acid) nor decreased BCFAs levels (isobutyric acid and isovaleric acid) (Figure S2), demonstrating the composition of gut bacteria varied very little after administration of SeCMP or CMP.

### 3.7. SeCMP Prevents Obesity-Driven Dysbiosis

**Figure 5:**
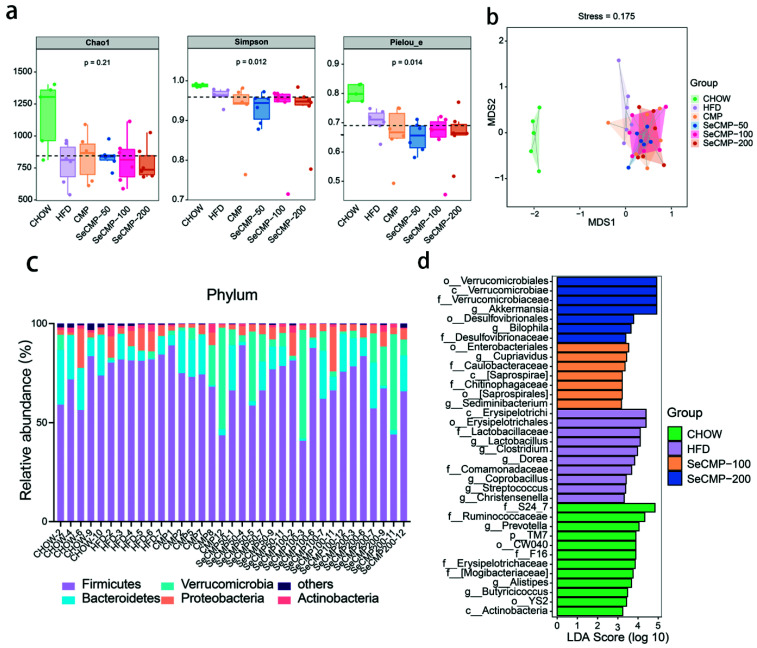
Difference in richness, diversity, and evenness of gut microbiota, relative abundance of bacteria at phylum level, and LEfSe analysis identified microbe biomarkers at genus level in mice fed with selenium-rich *C. militaris* crude polysaccharides (SeCMP) and selenium-deficient *C. militaris* crude polysaccharides (CMP). (**a**) Alpha diversity indexes of Chao1, Simpson, and Pielou’s evenness index. (**b**) Bray–Curtis distance based nonmetric multidimensional scaling (NMDS) (stress = 0.175). (**c**) Bacterial taxonomic profiling in the phylum level. (**d**) Main indicator taxa in each group based on LEfSe comparison (LDA score > 2). Data represent mean ± SE (n = 5–6 mice/group). CHOW: normal diet + sterile water; HFD: high-fat diet + sterile water; CMP: high-fat diet +100 mg/kg CMP; SeCMP-50: high-fat diet +50 mg/kg SeCMP; SeCMP-100: high-fat diet +100 mg/kg SeCMP; SeCMP-200: high-fat diet +200 mg/kg SeCMP.

High-throughput 16S rRNA sequencing was performed to analyze the host caecum feces bacteria community. Chao 1, Simpson, and Pielou parameters demonstrated that the diversity, richness, and evenness of the bacterial community in polysaccharides intake mice were not recovered to CHOW level (Figure 5a). Nonmetric multidimensional scaling (NMDS) based on the Bray–Curtis distance showed a drastic separation of the HFD group from the CHOW group (Figure 5b). SeCMP and CMP-treated mice clustered separately from HFD treated mice gut bacteria (stress value = 0.175), implying notable gut microbiota composition changes after polysaccharide administration (Figure 5b). No significant separation appeared between SeCMP and CMP treatment. Pair-wise ANOSIM and PERMANOVA tests indicated a significant separation among groups (*p* < 0.05) (Tables S3 and S4). The above results indicated that SeCMP and CMP supplementation altered beta diversity of obese mice, and their microbial community structure showed notable variations compared with the HFD group. In conclusion, SeCMP and CMP supplementation reshaped the gut microbiota composition, thus changing the host health status. Meanwhile, the richness, diversity, and entire structure of gut microbiota changed similarly under treatment with SeCMP-50, SeCMP-100, SeCMP-200, and CMP.

Phyla Firmicutes and Bacteroidetes were the major bacteria in the gut (Figure 5c). There was no significant difference among all the treatments on gut bacterial populations at the phylum level and Firmicutes/Bacteroidetes ratio (Figure S3a–f). At the genus level, under the LEfSe approach, Lactobacillus, Dorea, Clostridium, Christensenella, Coprobacillus, and Streptococcus appeared as the main featured gut microbiota community (Figure 5d), among which the relative abundance of Lactobacillus, Dorea, and Clostridium was prominently reduced by SeCMP intervention (Figure S4i–k). Akkermansia and Bilophila were considered as the key microbes of SeCMP-200 treated mice.

**Figure 6:**
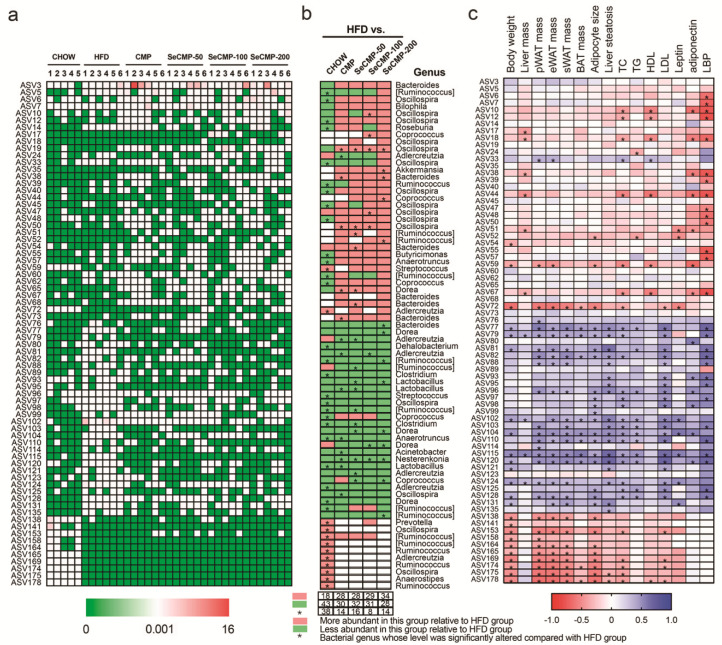
The relative abundance of specific amplicon sequence variants (ASV) identified at genus level and their association with obesity parameters affected by administration of selenium-rich *C. militaris* crude polysaccharides (SeCMP) and selenium-deficient *C. militaris* crude polysaccharides (CMP). (**a**) Heatmap of 72 identified bacterial genera affected by CHOW, HFD, CMP, SeCMP-50, SeCMP-100, and SeCMP-200 in comparison with the HFD group based on MetagenomeSeq analysis. (**b**) Bacterial genera from panel (**a**) and changes induced by the treatments. Red and green blocks indicated genera that were more or less abundant in the groups compared with the HFD group. Black stars indicated genera levels were significantly altered by HFD but reversed by treatments. (**c**) Correlation matrix between the identified ASVs and obesity traits using Pearson’s correlation analysis. * *p* < 0.05. CHOW: normal diet + sterile water; HFD: high-fat diet + sterile water; CMP: high-fat diet +100 mg/kg CMP; SeCMP-50: high-fat diet +50 mg/kg SeCMP; SeCMP-100: high-fat diet +100 mg/kg SeCMP; SeCMP-200: high-fat diet +200 mg/kg SeCMP.

Metagenomeseq analysis was used to identify the alternation of indicator ASVs by SeCMP and CMP (Figure 6a,b and Supplementary dataset). One hundred and seventy-nine ASVs were significantly affected by at least one intervention versus the HFD group, among which 72 ASVs were annotated at the genus level (Figure 6a,b). Pearson’s correlation analysis was performed to determine the correlation between the significantly altered gut microbiota biomarkers and specific physiological parameters relating to lipid disorder and inflammation (Figure 6c). Fifty-eight ASVs were notably correlated to at least one host dyslipidemia index. SeCMP and CMP administration increased 21 identified ASVs negatively correlated with disease phenotypes, such as Oscillospira, Akkermansia, Bacteroides, Coprococcus, and [Ruminococcus]. In addition, polysaccharide supplementation decreased 27 identified ASVs that were positively correlated with obesity traits, including Dorea, Adlercreutzia, [Ruminococcus], Lactobacillus, Clostridium, Anaerotruncus, Acinetobacter, Oscillospira, Nesterenkonia, and Coprococcus. In conclusion, taking the relative abundance into consideration, we decided to take Dorea, Lactobacillus, Clostridium, Ruminococcus, and Akkermansia ASVs as the key microbial phenotypes that potentially mediated the salutary effect of SeCMP and CMP on the HFD-induced obesity and hyperlipidemia.

## 4. Discussion

### 4.1. Intervention of SeCMP on Fat Accumulation and Hyperlipidemia

This study investigated the effect of naturally Se-containing polysaccharide isolated from an edible fungi *C. militaris* on HFD-induced obese mice and the associated metabolic alternations and gut microbiota profile. Prebiotic intake was considered to benefit the host’s health. Wu et al. [31] reported that 20 mg/kg bw administration of high molecular weight polysaccharides H1 (>300 kDa) extracted from wild *C. sinensis* reduced obesity and type-2 diabetes. In this study, we proved that a high dose of SeCMP decreased both peripheral and visceral WAT, liver lipid accumulation, and hyperlipidemia in obese mice. Hence, the obese suppressive effect of SeCMP ingestion was mainly due to the suppression of adiposity. In the previous work, we discovered that *C. militaris* mycelium, cultivated on rice medium, needed only 35 days to yield fruit bodies [32]. The decrement growth cycle largely saved the cost and time in obtaining raw materials. Hence, SeCMP administration would be a practical strategy to control HFD-induced obesity and maintain metabolic health.

Metabolically health is a new strategy to evaluate the risk of mortality and cardiovascular disease on obese individuals [33]. TC and LDL-C are risk factors for cardiovascular diseases, such as arteriosclerosis [34]. Previous studies had observed polysaccharide intake negatively correlated with serum TG, TC, and LDL-C levels [35]. Our result indicated a high dosage of SeCMP drastically reduced TG and LDL-C levels, mitigated hyperlipidemia, and benefitted the metabolically unhealthy obese individuals.

Se-rich natural products have good antioxidant and anti-inflammatory effects [36]. Previous studies have demonstrated the Se-fermented probiotics complex [14], Se-modified polysaccharide [15], and Se-rich black tea [20] can prevent body weight, improve lipid metabolism, and enhance the activities of antioxidant enzymes of hyperlipidemic mice. In this study, we found that mice treated with 100 mg/kg bw of SeCMP (Se content was 5.1 mg/kg) did not display a stronger effect on body weight and fat accumulation reduction compared with the same dose of CMP (Table 1), which was consistent with Gao et al.’s result [20]. The effect of Se in vivo depends on its dose and chemical structure [37]. Se content in Se-rich *C. militaris* fruit body in this work was still lower than other high-Se species. For example, *Pleurotus ostreatus* is able to accumulate Se up to 857 μg/g [38], and Se content in *Lentinus edodes* can reach 356 μg/g [39]. Hence, a higher level of Se concentration might appear better for biological activities.

CYP7A1 is a rate-limiting enzyme to BA synthesis in the liver [40]. Li et al. [41] observed GFP-H attenuating hepatic lipid and cholesterol levels of obese mice might be due to enhancing BA synthesis by upregulating *Cyp7a1* gene expression in the liver. We observed *Cyp7a1* gene expression was significantly upregulated in SeCMP intervention groups, which was consistent with Li et al.’s study. Hence, it is possible that SeCMP accelerates the transfer of cholesterol to BAs in obese hosts.

Obesity is characterized by increasing serum levels of pro-inflammatory cytokines, secreting particularly from visceral adipose tissue. Leptin is an adipocyte hormone to regulate appetite, and its amount is in proportion to the mass of adipose tissue [42]. M1-polarized macrophages around dying WAT increased the release of TNF-*α* and IL-6, resulting in a local and systemic low-grade inflammation [43]. Adiponectin is an anti-inflammatory and insulin-sensitizing adipokine [44]. Moreover, the translocation of LPS over the gut barrier from gut microbiota also played a role in low-grade inflammation. LPS primarily acted as an agonist to TLR4 binding by LBP; the complex triggers a cascade of inflammatory cytokine expression, production, and secretion. Interestingly, the notable reduction in leptin, adiponectin, LBP, TNF-*α,* and IL-6 levels observed in the SeCMP-200 group and the increase in IL-10 in fat implies a lower WAT and appetite effect of SeCMP-200 feeding mice compared to the HFD group (Figure 3 and Figure 4).

In adipocytes, the thermogenic function is mediated largely by activating UCP1 for energy expenditure instead of producing ATP [45]. Zhang et al. [46] reported that phytochemical berberine not only induced UCP1 expression in BAT but also promoted the differentiation of WAT into BAT. Therefore, it further contributed to thermogenesis and weight loss. In this study, the dramatic increase in UCP1 expression was also observed in BAT but not in eWAT. The subcutaneous inguinal WAT in mice induced profound amounts of beige adipocytes, whereas the eWAT of male mice was particularly resistant to “beige-ing” [47]. Hence, these results suggested when obese mice were administrated with SeCMP-200, its elevated expression of UCP1 might improve the lipid deposition decrement through energy expenditure.

Recent reports demonstrated the structure of *C. militaris* polysaccharides was composed of (1➝2)-*α*-D-Manp and (1➝4)-*α*-D-Glcp and *β*-D-glucan as its backbone [7,9]. Since digestive enzymes secreted by the pancreas cannot hydrolyze *β*-glucosidic bonds, and these non-digestible carbohydrate rich in *β*-glucosidic bonds could escape from gastrointestinal tract digestion and therefore be utilized by gut microorganisms to produce SCFAs, which are greatly beneficial to the host [48]. In this study, SCFAs concentration in all polysaccharide treatments was not notably changed compared with HFD-fed mice. It might be attributed to less *β*-glucosidic bond content in *C. militaris* polysaccharides.

It was reported [49,50] that dietary fibers facilitated the growth of enteric microbes and the production of large amounts of liner-chain fatty acids to benefit the host. When fermentable fibers are in short supply, microbes switch to energetically less favorable sources for growth, such as amino acids, endogenous protein, or dietary fat. Protein fermentation can contribute to the SCFA pool, but it mostly gives rise to detrimental microbial metabolites—branched-chain fatty acids (BCFAs), which are associated with insulin resistance. The bacteria metabolites SCFAs and BCFAs contents did not change after all the polysaccharides treatments, suggesting they did not contribute to obesity prevention.

### 4.2. CMP Alleviated the HFD-Induced Obesity via Modifying Gut Microbiota Composition

There was no significant difference in bacterial alpha and beta diversity between all the polysaccharides treatments and HFD supplementation (Figure 5a,b). However, some health-promoting varieties, such as *Akkermansia,* appeared in the SeCMP-200 group, and the detrimental bacteria harmful to the host decreased, which was consistent with previous reports [51]. The modification of genus or species level may benefit host health, as reported by Wu et al. [31].

Multivariate statistical analysis of caecum bacteria revealed dietary intervention reshaped the HFD-fed mice gut microbiota. SeCMP and CMP profoundly reduced the genus *Lactobacillus*. It was also observed in the CC-treat mice [52]. The relative abundance of *Lactobacillus* was strongly correlated with serum cholesterol, triglyceride, and LDL-C level. BA pool size is positively associated with the expansion of BA-resistant bacteria (e.g., *Lactobacillus* spp.). Anhê et al. [52] attributed the decrease in the relative abundance of *Lactobacillus* to the reduction in BA pool size. In addition, intake of phytochemicals may restrain the lumen oxygen availability and further contributing to the bloom of facultative anaerobe species, such as *Lactobacilli* [53]. Moreover, the relative abundance of a series pathogen *Clostridium*, *Ruminococcus,* and *Dorea* decreased, which was in line with the previous result [54,55,56]. These microbes were positively correlated with the lipid metabolism and inflammation parameters in the host. For example, Tang et al. [54] showed that chitosan administration increased the anti-obesity-related species *C. leptum*, and significantly decreased *C. lactatefermentans* and *C. cocleatum*. *Ruminococcus* and *Dorea* were common in IBD or colitic individuals, which were strongly correlated with inflammatory cytokines, such as TNF-*α*. These microbes might act as pro-inflammatory factors aggravating obesity progress [55,56].

*Akkermansia* was the emerging key genus identified in the SeCMP-200 group by LEfSe analysis. *A. muciniphila* is regarded as the next-generation beneficial microbe because it prevents the development of obesity, diabetes, and the associated low-grade inflammation [57]. It is a mucosal parasite that may utilize host-derived mucins. Administration of *A. muciniphila* to mice restored the normal mucus layer thickness and produced some antimicrobial peptides to prevent the development of metabolic endotoxemia [58]. AMUC 1100, the most abundant membrane protein expressed on *A. muciniphila’s* outer membrane, was proved stable after pasteurization. This method unexpectedly exacerbated the microbe’s beneficial impact on the host by decreasing energy absorption, improving insulin sensitivity, improving gut barrier, and completely blocking the diet-induced metabolic endotoxemia [59]. Hence, a high dose of SeCMP prompted the growth of probiotics, explaining why the beta diversity of SeCMP-200 was different from the CHOW group.

It has been well recognized that the gut microbiota has a great impact on the host metabolic status [60]. Some dietary fibers, phytochemicals, vitamins, and minerals could regulate some specific groups of bacteria and promote intestinal colonization. Recent work performed by Zhai et al. [18] found different sodium selenite supplementation did not significantly alter the overall richness of the gut microbiota in mice, but it decreased the level of *Dorea* and biomarkers related to inflammation, including IL-1*β*, IL-6, IL-8, TNF-*α*; in addition, the supplementation increased the *Akkermansia* level and tight junction gene of *Zo1*, claudin-1, and occludin, which would benefit the intestinal barrier and immune responses. Oral administration of *Grifola frondosa* polysaccharides affected serum lipid level, fat accumulation, and lipid metabolism by reducing lipid profiles negatively correlated gut microbiota phylotypes *Ruminococcus*, enhancing *Cyp7a1* gene expression and BA [41]. Our observations were in line with these results, suggesting SeCMP stimulated the modification of intestinal microbes and intestinal immune response.

In this study, a rather high fatty feeding (60%) was adopted to build the obesity model. High fat in the diet (fat content at 24–45%) could induce high calories intolerance [61]. A moderate-fat diet administration assay might be helpful in understanding the internal action of functional food. On the other hand, omics can be involved to study the metabolic mechanism in detail in the future. For example, liver transcriptomics, metabonomics, and composition of BAs in feces would disclose the molecular mechanism of the lipid metabolism process, for we assumed SeCMP and CMP might affect cholesterol transformation in the BA pathway in this work. In addition, some physiological indices, including the activity, behavior, energy expenditure, oxygen consumption, carbon dioxide production, and body temperature [52,62], will be monitored to further clarify the thermogenesis ability of SeCMP intake.

In conclusion, we isolated two crude polysaccharides SeCMP and CMP, using a simple and quick method in this work. Although SeCMP did not show superiority to CMP administration in treating HFD-induced obesity, high-dose administration of SeCMP was proved to ameliorate fat accretion, dyslipidemia, inflammation, and gut microbiota dysbiosis in obese mice. SeCMP might be a therapeutic tool in the management of lipid metabolic disorders. The development of SeCMP-rich functional foods for lipid disorder seems to be a promising alternative remedy.

## Figures and Tables

**Table 1:** Chemical compositions of crude polysaccharides isolated from Se-deficient C. *militaris* (CMP) and Se-rich C. *militaris* (SeCMP).

	CMP	SeCMP
Se (mg/kg)	0.10 ± 0.01	5.14 ± 0.16 *
Dietary fiber (g/100 g)	12.4 ± 0.25	8.8 ± 0.17 *
Carbohydrate (g/100 g)	37.8 ± 0.13	45.0 ± 0.16 *
Crude polysaccharides (g/100 g)	47.4 ± 2.02	56.7 ± 3.91 *
Protein (g/100 g)	35.1 ± 0.01	31.1 ± 0.15 *
Lipid (g/100 g)	1.2 ± 0.15	1.3 ± 0.15
Hydrolyzed amino acids (g/100 g)	20.8 ± 0.36	18.5 ± 0.29 *
Phenols (g/100 g)	0.21 ± 0.01	0.17 ± 0.01 *
Energy (kcal/100 g)	302.2 ± 0.83	316.3 ± 1.33 *

Se: selenium. Data are presented as mean ± SE (*n* = 3). * Indicated significant variation between the values within a row; *p* < 0.05.

**Table 2:** Weight of liver and adipose tissues of mice under different feeding treatments.

Group	Liver (g)	Perirenal Fat (g)	Epididymal Fat (g)	Subcutaneous Fat (g)	BAT (g)
CHOW	1.041 ± 0.077 ^b^	0.123 ± 0.065 ^a^	0.459 ± 0.147 ^a^	0.255 ± 0.081 ^a^	0.134 ± 0.032 ^a^
HFD	0.832 ± 0.058 ^a^	0.718 ± 0.221 ^c^	1.805 ± 0.490 ^c^	1.107 ± 0.357 ^c^	0.191 ± 0.057 ^b^
CMP	0.821 ± 0.071 ^a^	0.604 ± 0.210 ^bc^	1.404 ± 0.435 ^bc^	0.821 ± 0.299 ^bc^	0.154 ± 0.038 ^ab^
SeCMP-50	0.782 ± 0.074 ^a^	0.535 ± 0.115 ^bc^	1.331 ± 0.334 ^bc^	0.887 ± 0.227 ^bc^	0.169 ± 0.032 ^ab^
SeCMP-100	0.755 ± 0.073 ^a^	0.628 ± 0.263 ^bc^	1.373 ± 0.312 ^bc^	1.013 ± 0.298 ^bc^	0.152 ± 0.032 ^ab^
SeCMP-200	0.779 ± 0.064 ^a^	0.429 ± 0.204 ^b^	1.092 ± 0.451 ^b^	0.601 ± 0.258 ^b^	0.150 ± 0.027 ^ab^

CHOW: normal diet + sterile water; HFD: high-fat diet + sterile water; CMP: high-fat diet +100 mg/kg Se-deficient *C. militaris* crude polysaccharides; SeCMP-50: high-fat diet +50 mg/kg Se-rich *C. militaris* crude polysaccharides; SeCMP-100: high-fat diet +100 mg/kg Se-rich *C. militaris* crude polysaccharides; SeCMP-200: high-fat diet +200 mg/kg Se-rich *C. militaris* crude polysaccharides; BAT: brown adipocyte tissue. Data are expressed as mean ± SE (n = 8 mice/group). Superscript characters indicate significant variation between different values within a column.

## Supplementary Materials

The following are available online at https://www.mdpi.com/article/10.3390/foods10102252/s1, Figure S1: Polysaccharides supplementation do not affect energy intake, water intake, and energy extraction, Figure S2: Selenium-deficient *C. militaris* crude polysaccharides (CMP) and selenium-deficient *C. militaris* crude polysaccharides (SeCMP) do not affect the production of short-chain fatty acids, Figure S3: The relative abundance of top 5 phyla and F/B ratio after polysaccharides administration, Figure S4: The relative abundance of top 10 genera and key genera identified in LEfSe comparsion test, Table S1: Weight of organs of mice under different feeding treatment, Table S2: Biochemistry analysis of serum of mice, Table S3: Group significance using ANOSIM analysis based on Bray–Curtis distance, Table S4: Group significance by PERMANOVA analysis based on Bray–Curtis distance; Table S5: Q-RCP primer sequences; Supplementary dataset (Microsoft Excel file): Relative abundance of bacterial species showing differences between the HFD-treated group and CHOW/CMP/SeCMP-intervened groups after feeding for 8 weeks (related to Figure 5).

## References

[1] GBD 2015 Obesity Collaborators (2017). Health Effects of Overweight and Obesity in 195 Countries over 25 Years. N. Engl. J. Med..

[2] Yanovski S.Z., Yanovski J.A. (2014). Long-term Drug Treatment for Obesity: A Systematic and Clinical Review. JAMA.

[3] Newman C.B., Preiss D., Tobert J.A., Jacobson T.A., Page II R.L., Goldstein L.B., Chin C., Tannock L.R., Miller M., Raghuveer G. (2019). Statin Safety and Associated Adverse Events: A Scientific Statement from the American Heart Association. Arterioscler. Thromb. Vasc. Biol..

[4] Bray G.A., Frühbeck G., Ryan D.H., Wilding J.P.H. (2016). Management of obesity. Lancet.

[5] Yu R., Ye B., Yan C., Song L., Zhang Z., Yang W., Zhao Y. (2007). Fingerprint analysis of fruiting bodies of cultured *Cordyceps militaris* by high-performance liquid chromatography-photodiode array detection. J. Pharm. Biomed. Anal..

[6] Wu L., Sun H., Hao Y., Zheng X., Song Q., Dai S., Zhu Z. (2020). Chemical structure and inhibition on α-glucosidase of the polysaccharides from *Cordyceps militaris* with different developmental stages. Int. J. Biol. Macromol..

[7] He B.-L., Zheng Q.-W., Guo L.-Q., Huang J.-Y., Yun F., Huang S.-S., Lin J.-F. (2020). Structural characterization and immune-enhancing activity of a novel high-molecular-weight polysaccharide from *Cordyceps militaris*. Int. J. Biol. Macromol..

[8] Bi S., Jing Y., Zhou Q., Hu X., Zhu J., Guo Z., Song L., Yu R. (2018). Structural elucidation and immunostimulatory activity of a new polysaccharide from *Cordyceps militaris*. Food Funct..

[9] Zhang Y., Zeng Y., Cui Y., Liu H., Dong C., Sun Y. (2020). Structural characterization, antioxidant and immunomodulatory activities of a neutral polysaccharide from *Cordyceps militaris* cultivated on hull-less barley. Carbohydr. Polym..

[10] Zhu Z.-Y., Guo M.-Z., Liu F., Luo Y., Chen L., Meng M., Wang X.-T., Zhang Y.-M. (2016). Preparation and inhibition on α-d-glucosidase of low molecular weight polysaccharide from *Cordyceps militaris*. Int. J. Biol. Macromol..

[11] Ji Y., Su A., Ma G., Tao T., Fang D., Zhao L., Hu Q. (2020). Comparison of bioactive constituents and effects on gut microbiota by in vitro fermentation between *Ophicordyceps sinensis* and *Cordyceps militaris*. J. Funct. Foods.

[12] Milovanovic I., Lajin B., Braeuer S., Steiner O., Lisa F., Goessler W. (2019). Simultaneous selenium and sulfur speciation analysis in cultivated *Pleurotus pulmonarius* mushroom. Food Chem..

[13] Labunskyy V.M., Hatfield D.L., Gladyshev V.N. (2014). Selenoproteins: Molecular pathways and physiological roles. Physiol. Rev..

[14] Zhao D., Gao F., Zhu H., Qian Z., Mao W., Yin Y., Chen D. (2020). Selenium-enriched *Bifidobacterium longum* DD98 relieves metabolic alterations and liver injuries associated with obesity in high-fat diet-fed mice. J. Funct. Foods.

[15] Surhio M.M., Wang Y., Xu P., Shah F., Li J., Ye M. (2017). Antihyperlipidemic and hepatoprotective properties of selenium modified polysaccharide from *Lachnum* sp.. Int. J. Biol. Macromol..

[16] Saxena A.J.D. Adiponectin and Selenium Rich Diet can Act as a Complimentary MEDICINE in the Treatment of Intestinal and Chronic Inflammation Induced Colon Cancer. https://scholarcommons.sc.edu/etd/3647/.

[17] Ferreira R.L.U., Sena-Evangelista K.C.M., de Azevedo E.P., Pinheiro F.I., Cobucci R.N., Pedrosa L.F.C. (2021). Selenium in Human Health and Gut Microflora: Bioavailability of Selenocompounds and Relationship with Diseases. Front. Nutr..

[18] Zhai Q., Cen S., Li P., Tian F., Zhao J., Zhang H., Chen W. (2018). Effects of Dietary Selenium Supplementation on Intestinal Barrier and Immune Responses Associated with Its Modulation of Gut Microbiota. Environ. Sci. Technol. Lett..

[19] Kasaikina M.V., Kravtsova M.A., Lee B.C., Seravalli J., Peterson D.A., Walter J., Legge R., Benson A.K., Hatfield D.L., Gladyshev V.N. (2011). Dietary selenium affects host selenoproteome expression by influencing the gut microbiota. FASEB J..

[20] Gao Y., Xu Y., Ruan J., Yin J. (2020). Selenium affects the activity of black tea in preventing metabolic syndrome in high-fat diet-fed Sprague–Dawley rats. J. Sci. Food Agric..

[21] DuBois M., Gilles K.A., Hamilton J.K., Rebers P.A., Smith F. (1956). Colorimetric method for determination of sugars and related substances. Anal. Chem..

[22] Kjeldahl J. (1883). A New Method for the Determination of Nitrogen in Organic Matter. Z. Anal. Chem..

[23] Luque-García J.L., Luque de Castro M.D. (2004). Ultrasound-assisted Soxhlet extraction: An expeditive approach for solid sample treatment: Application to the extraction of total fat from oleaginous seeds. J. Chromatogr. A.

[24] Gilani G., Peace R. (2005). Chromatographic determination of amino acids in foods. J. AOAC Int..

[25] Li S., Li J., Zhu Z., Cheng S., He J., Lamikanra O. (2020). Soluble dietary fiber and polyphenol complex in lotus root: Preparation, interaction and identification. Food Chem..

[26] Sun J., Yao J., Huang S., Long X., Wang J., García-García E. (2009). Antioxidant activity of polyphenol and anthocyanin extracts from fruits of *Kadsura coccinea* (Lem.) A.C. Smith. Food Chem..

[27] Pradeep C.R., Kuttan G. (2004). Piperine is a potent inhibitor of nuclear factor-kB (NF-kB), c-Fos, CREB, ATF-2 and proinflammatory cytokine gene expression in B16F-10 melanoma cells. Int. Immunopharmacol..

[28] Kraus D., Yang Q., Kahn B.B. (2015). Lipid Extraction from Mouse Feces. Bio-protocol.

[29] DeSantis T.Z., Hugenholtz P., Larsen N., Rojas M., Brodie E.L., Keller K., Huber T., Dalevi D., Hu P., Andersen G.L. (2006). Greengenes, a Chimera-Checked 16S rRNA Gene Database and Workbench Compatible with ARB. Appl. Environ. Microbiol..

[30] Zhou S., Wang Y., Jacoby J.J., Jiang Y., Zhang Y., Yu L.L. (2017). Effects of Medium- and Long-Chain Triacylglycerols on Lipid Metabolism and Gut Microbiota Composition in C57BL/6J Mice. J. Agric. Food Chem..

[31] Wu T.R., Lin C.S., Chang C.J., Lin T.L., Martel J., Ko Y.F., Ojcius D.M., Lu C.C., Young J.D., Lai H.-C. (2018). Gut commensal *Parabacteroides goldsteinii* plays a predominant role in the anti-obesity effects of polysaccharides isolated from *Hirsutella sinensis*. Gut.

[32] Yuan M. (2013). Artificial Cultivation and Active Ingredients Analysis of Cordyceps Militaris. Master’s Thesis.

[33] Ahima R.S., Lazar M.A. (2013). The Health Risk of Obesity—Better Metrics Imperative. Science.

[34] Sandesara P.B., Virani S.S., Fazio S., Shapiro M.D. (2018). The Forgotten Lipids: Triglycerides, Remnant Cholesterol, and Atherosclerotic Cardiovascular Disease Risk. Endocr. Rev..

[35] Yang X., Mo W., Zheng C., Li W., Tang J., Wu X. (2020). Alleviating effects of noni fruit polysaccharide on hepatic oxidative stress and inflammation in rats under a high-fat diet and its possible mechanisms. Food Funct..

[36] Rayman M.P. (2012). Selenium and human health. Lancet.

[37] Wang L., Huang Q., Yan F., Xie J., Qu C., Chen J., Zheng L., Yi T., Zenga H.-F., Li H. (2018). Comparison of protective effect of ordinary Cordyceps militaris and selenium-enriched Cordyceps militaris on triptolide-induced acute hepatotoxicity and the potential mechanisms. J. Funct. Foods.

[38] Silva M.C.S.d., Naozuka J., Luz J.M.R.d., Assunção L.S.d., Oliveira P.V., Vanetti M.C.D., Bazzolli M.S.D., Kasuy M.C.M. (2012). Enrichment of *Pleurotus ostreatus* mushrooms with selenium in coffee husks. Food Chem..

[39] Ogra Y., Ishiwata K., Ruiz Encinar J., Łobiński R., Suzuki K.T. (2004). Speciation of selenium in selenium-enriched shiitake mushroom, *Lentinula edodes*. Anal. Bioanal. Chem..

[40] Wahlström A., Sayin S.I., Marschall H.-U., Bäckhed F. (2016). Intestinal Crosstalk between Bile Acids and Microbiota and Its Impact on Host Metabolism. Cell Metab..

[41] Li L., Guo W.-L., Zhang W., Xu J.-X., Qian M., Bai W.-D., Zhang Y.-Y., Rao P.-F., Ni L., Lv X.-C. (2019). *Grifola frondosa* polysaccharides ameliorate lipid metabolic disorders and gut microbiota dysbiosis in high-fat diet fed rats. Food Funct..

[42] Polyzos S.A., Kountouras J., Mantzoros C.S. (2015). Leptin in nonalcoholic fatty liver disease: A narrative review. Metabolism.

[43] Kern L., Mittenbühler M.J., Vesting A.J., Ostermann A.L., Wunderlich C.M., Wunderlich F.T. (2019). Obesity-Induced TNFα and IL-6 Signaling: The Missing Link between Obesity and Inflammation—Driven Liver and Colorectal Cancers. Cancers.

[44] Hersoug L.G., Møller P., Loft S. (2016). Gut microbiota-derived lipopolysaccharide uptake and trafficking to adipose tissue: Implications for inflammation and obesity. Obes. Rev..

[45] Kajimura S., Spiegelman B.M., Seale P. (2015). Brown and Beige Fat: Physiological Roles beyond Heat Generation. Cell Metab..

[46] Zhang Z., Zhang H., Li B., Meng X., Wang J., Zhang Y., Yao S., Ma Q., Jin L., Yang J. (2014). Berberine activates thermogenesis in white and brown adipose tissue. Nat. Commun..

[47] Ohno H., Shinoda K., Spiegelman B.M., Kajimura S. (2012). PPARγ agonists Induce a White-to-Brown Fat Conversion through Stabilization of PRDM16 Protein. Cell Metab..

[48] Friedman M. (2016). Mushroom Polysaccharides: Chemistry and Antiobesity, Antidiabetes, Anticancer, and Antibiotic Properties in Cells, Rodents, and Humans. Foods.

[49] Koh A., De Vadder F., Kovatcheva-Datchary P., Bäckhed F. (2016). From Dietary Fiber to Host Physiology: Short-Chain Fatty Acids as Key Bacterial Metabolites. Cell.

[50] Sanna S., van Zuydam N.R., Mahajan A., Kurilshikov A., Vich Vila A., Võsa U., Mujagic Z., Masclee A.A.M., Jonkers D.M.A.E., Oosting M. (2019). Causal relationships among the gut microbiome, short-chain fatty acids and metabolic diseases. Nat. Genet..

[51] Chen J., Liu J., Yan C., Zhang C., Pan W., Zhang W., Lu Y., Chen L., Chen Y. (2020). *Sarcodon aspratus* polysaccharides ameliorated obesity-induced metabolic disorders and modulated gut microbiota dysbiosis in mice fed a high-fat diet. Food Funct..

[52] Anhê F.F., Nachbar R.T., Varin T.V., Trottier J., Dudonné S., Le Barz M., Feutry P., Pilon G., Barbier O., Desjardins Y. (2019). Treatment with camu camu (*Myrciaria dubia*) prevents obesity by altering the gut microbiota and increasing energy expenditure in diet-induced obese mice. Gut.

[53] Li F., Jiang C., Krausz K.W., Li Y., Albert I., Hao H., Fabre K.M., Mitchell J.B., Patterson A.D., Gonzalez F.J. (2013). Microbiome remodelling leads to inhibition of intestinal farnesoid X receptor signalling and decreased obesity. Nat. Commun..

[54] Tang D., Wang Y., Kang W., Zhou J., Dong R., Feng Q. (2020). Chitosan attenuates obesity by modifying the intestinal microbiota and increasing serum leptin levels in mice. J. Funct. Foods.

[55] Han Y., Song M., Gu M., Ren D., Zhu X., Cao X., Li F., Wang W., Cai X., Yuan B. (2019). Dietary Intake of Whole Strawberry Inhibited Colonic Inflammation in Dextran-Sulfate-Sodium-Treated Mice via Restoring Immune Homeostasis and Alleviating Gut Microbiota Dysbiosis. J. Agric. Food Chem..

[56] Raza G.S., Maukonen J., Makinen M., Niemi P., Niiranen L., Hibberd A.A., Poutanen K., Buchert J., Herzig K.-H. (2019). Hypocholesterolemic Effect of the Lignin-Rich Insoluble Residue of Brewer’s Spent Grain in Mice Fed a High-Fat Diet. J. Agric. Food Chem..

[57] Cani P.D., de Vos W.M. (2017). Next-Generation Beneficial Microbes: The Case of *Akkermansia muciniphila*. Front. Microbiol..

[58] Everard A., Belzer C., Geurts L., Ouwerkerk J.P., Druart C., Bindels L.B., Guiot Y., Derrien M., Muccioli G.G., Delzenne N.M. (2013). Cross-talk between *Akkermansia muciniphila* and intestinal epithelium controls diet-induced obesity. Proc. Natl. Acad. Sci. USA.

[59] Plovier H., Everard A., Druart C., Depommier C., Van Hul M., Geurts L., Chilloux J., Ottman N., Duparc T., Lichtenstein L. (2017). A purified membrane protein from *Akkermansia muciniphila* or the pasteurized bacterium improves metabolism in obese and diabetic mice. Nat. Med..

[60] Sonnenburg J.L., Bäckhed F. (2016). Diet–microbiota interactions as moderators of human metabolism. Nature.

[61] Huang H., Jiang X., Xiao Z., Yu L., Pham Q., Sun J., Chen P., Yokoyama W., Yu L.L., Luo Y.S. (2016). Red Cabbage Microgreens Lower Circulating Low-Density Lipoprotein (LDL), Liver Cholesterol, and Inflammatory Cytokines in Mice Fed a High-Fat Diet. J. Agric. Food Chem..

[62] Wang B., Kong Q., Li X., Zhao J., Zhang H., Chen W., Wang G. (2020). A High-Fat Diet Increases Gut Microbiota Biodiversity and Energy Expenditure Due to Nutrient Difference. Nutrients.

