# Supplementary material for: Anti-Hyperlipidemia and Gut Microbiota Community Regulation Effects of Selenium-Rich Cordyceps militaris Polysaccharides on the High-Fat Diet-Fed Mice Model
Source: Foods. 2021 Sep 23;10(10):2252. doi: 10.3390/foods10102252 (PMC8534605; doi:10.3390/foods10102252)
Supplement: Supplementary file 1 [file foods-10-02252-s001.zip › Supporting Information.pdf]

Supplementary Materials

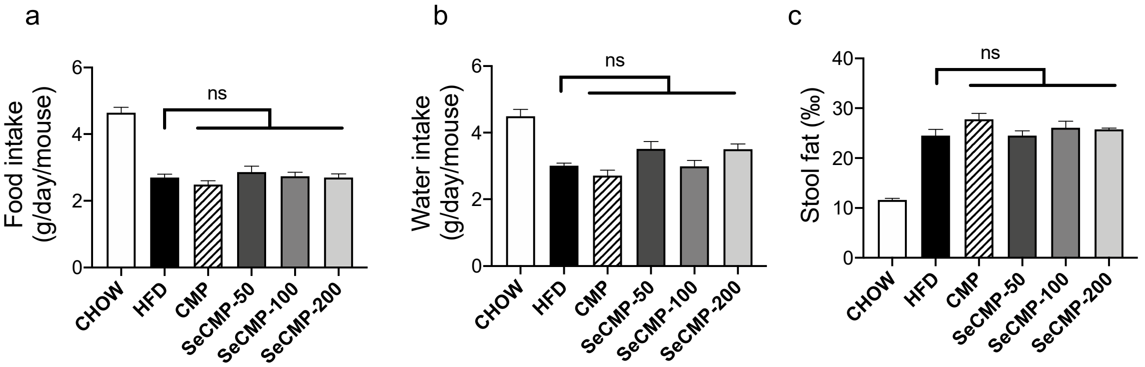

Figure S1. Polysaccharides supplementation do not affect energy intake, water intake and energy extraction. (a) Food intake and (b) water intake were determined once per week of each cage and converted to daily intake per mouse. (c) Stool fat was assessed by gravimetric analysis. Data are expressed as mean ± SE (n = 8 mice/group). Data were analyzed using one-way ANOVA analysis with Tukey's post hoc test or the non-parametric Kruskal-Wallis test with Dunnett's T3 multiple comparison test; ns represents not significant. CHOW: normal diet + sterile water; HFD: high-fat diet + sterile water; CMP: high-fat diet + 100 mg/kg selenium-deficient *C. militaris* crude polysaccharides; SeCMP-50: high-fat diet + 50 mg/kg SeCMP; SeCMP-100: high-fat diet + 100 mg/kg SeCMP; SeCMP-200: high-fat diet + 200 mg/kg SeCMP.

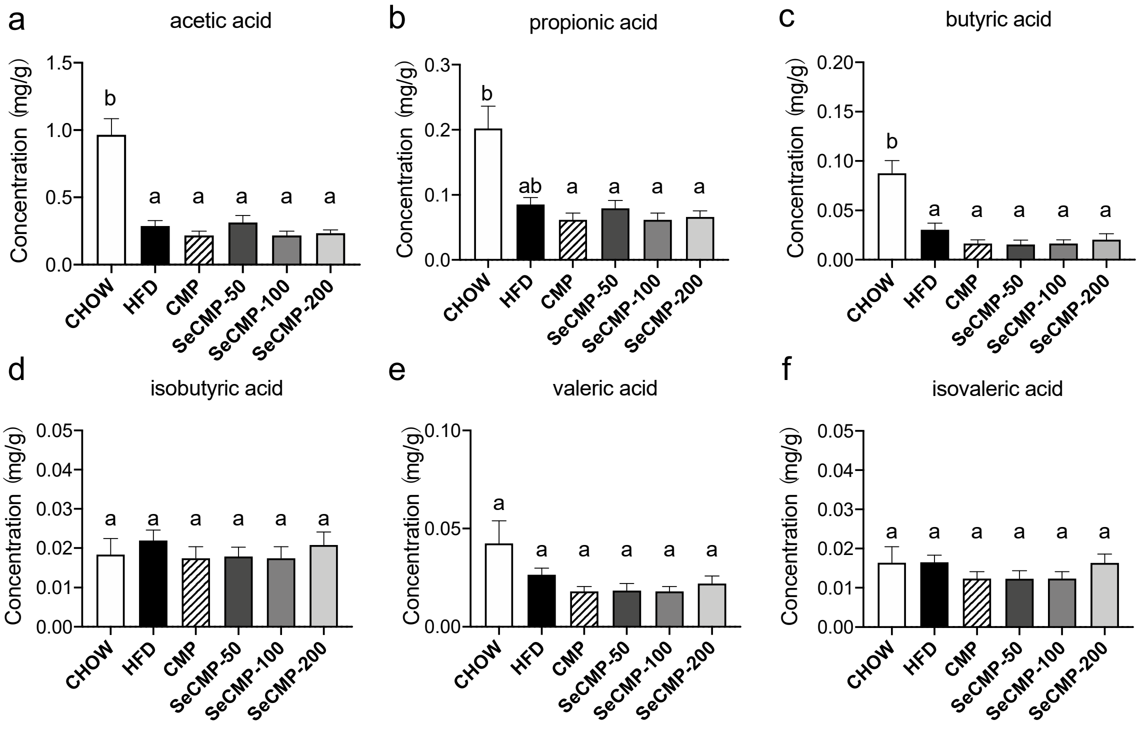

Figure S2. Selenium-deficient *C. militaris* crude polysaccharides (CMP) and selenium-deficient *C. militaris* crude polysaccharides (SeCMP) do not affect production of short chain fatty acids. Linear chain of (a) acetate, (b) propionate, (c) butyrate and (e) valeric acid and branched chain (d) isobutyric acid and (f) isovaleric acid were quantified using GC-MS. Data represent mean ± SE (n = 8 mice/group). Data were analyzed using Tukey's post hoc test or the non-parametric Kruskal-Wallis test with Dunnett's T3 multiple comparison test. Superscript characters indicate significant variation among treatments. CHOW: normal diet + sterile water; HFD: high-fat diet + sterile water; CMP: high-fat diet + 100 mg/kg CMP; SeCMP-50: high-fat diet + 50 mg/kg SeCMP; SeCMP-100: high-fat diet + 100 mg/kg SeCMP; SeCMP-200: high-fat diet + 200 mg/kg SeCMP.

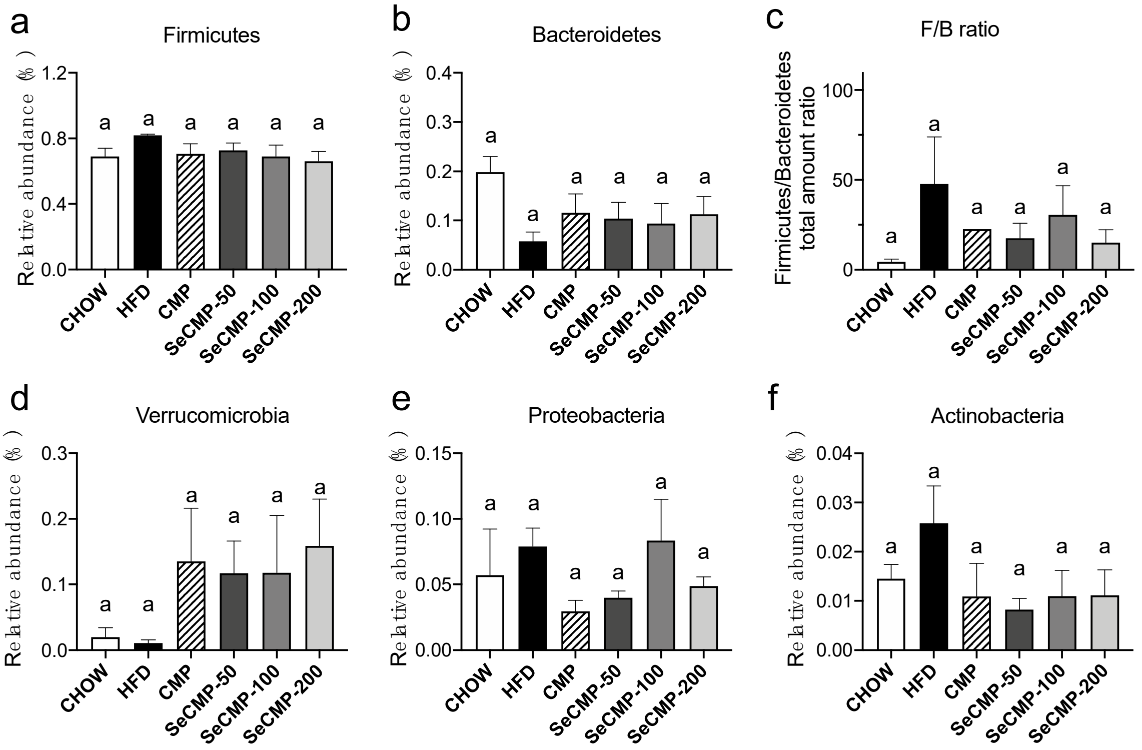

Figure S3. The relative abundance of top 5 phyla and F/B ratio, including (a) Firmicutes, (b) Bacteroidetes, (c) Firmicutes/Bacteroidetes ratio, (d) Verrucomicrobia, (e) Proteobacteria and (f) Actinobacteria after polysaccharides administration. Data represent mean ± SE (*n* = 5-6 mice/group). Data were analyzed using Tukey's post hoc test or the non-parametric Kruskal-Wallis test with Dunnett's T3 multiple comparison test. Superscript characters indicate significant variation among treatments. CHOW: normal diet + sterile water; HFD: high-fat diet + sterile water; CMP: high-fat diet + 100 mg/kg CMP; SeCMP-50: high-fat diet + 50 mg/kg SeCMP; SeCMP-100: high-fat diet + 100 mg/kg SeCMP; SeCMP-200: high-fat diet + 200 mg/kg SeCMP.

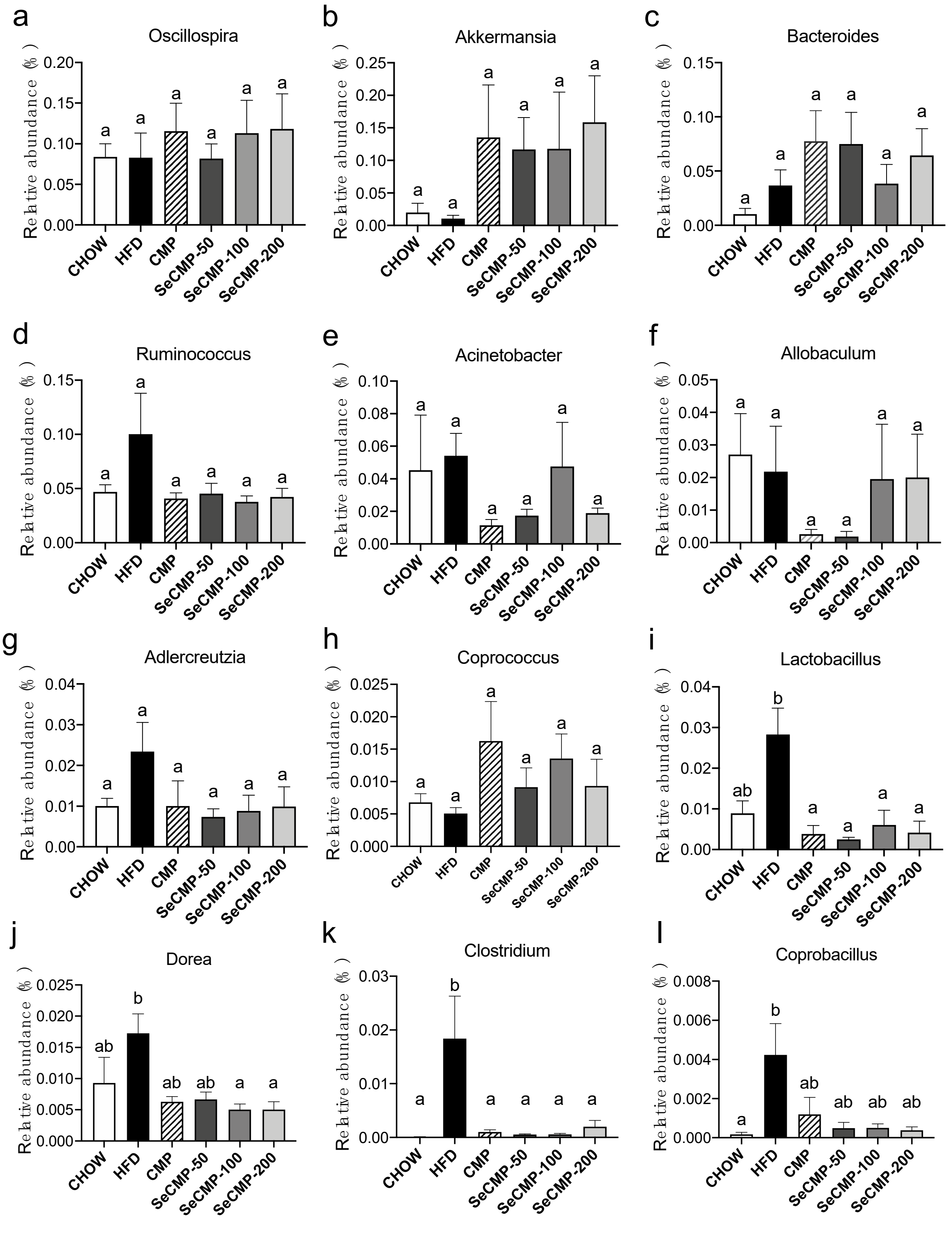

Figure S4. The relative abundance of top 10 genera and key genera identified in LEfSe comparsion test. Data represent mean ± SE (*n* = 5-6 mice/group). Data were analyzed using Tukey's post hoc test or the non-parametric Kruskal-Wallis test with Dunnett's T3 multiple comparison test. Superscript characters indicate significant variation among treatments. CHOW: normal diet + sterile water; HFD: high-fat diet + sterile water; CMP: high-fat diet + 100 mg/kg CMP; SeCMP-50: high-fat diet + 50 mg/kg SeCMP; SeCMP-100: high-fat diet + 100 mg/kg SeCMP; SeCMP-200: high-fat diet + 200 mg/kg SeCMP.

Table S1. Weight of organs of mice under different feeding treatment.

	Pancreas (g)	Heart (g)	Spleen (g)	Lung (g)	Kidney (g)
CHOW	0.108±0.005^{a}	0.150±0.007^{a}	0.091±0.007^{b}	0.166±0.004^{b}	0.352±0.018^{b}
HFD	0.114±0.004^{a}	0.129±0.006^{a}	0.064±0.004^{a}	0.142±0.006^{ab}	0.274±0.008^{a}
CMP	0.129±0.008^{a}	0.122±0.007^{a}	0.076±0.008^{ab}	0.139±0.006^{a}	0.262±0.009^{a}
SeCMP-50	0.116±0.009^{a}	0.128±0.005^{a}	0.068±0.004^{ab}	0.140±0.005^{a}	0.264±0.006^{a}
SeCMP-100	0.108±0.005^{a}	0.124±0.008^{a}	0.068±0.007^{ab}	0.142±0.009^{a}	0.258±0.008^{a}
SeCMP-200	0.118±0.008^{a}	0.130±0.007^{a}	0.075±0.007^{ab}	0.143±0.006^{ab}	0.273±0.008^{a}

Data are expressed as mean ± SE (n = 8 mice/group). Data were analyzed using one-way ANOVA analysis with Tukey's post hoc test or the non-parametric Kruskal-Wallis test with Dunnett's T3 multiple comparison test. Superscript characters indicate significant variation between different values within a column. CHOW: normal diet + sterile water; HFD: high-fat diet + sterile water; CMP: high-fat diet + 100 mg/kg selenium-deficient *C. militaris* crude polysaccharides; SeCMP-50: high-fat diet + 50 mg/kg SeCMP; SeCMP-100: high-fat diet + 100 mg/kg SeCMP; SeCMP-200: high-fat diet + 200 mg/kg SeCMP.

Table S2. Biochemistry analysis of serum of mice.

	CHOW	HFD	CMP	SeCMP-50	SeCMP-100	SeCMP-200
**ALT (IU/L)**	29.05±1.45[a]	28.64±1.42[a]	33.07±5.54[a]	26.17±1.04[a]	28.50±1.98[a]	25.88±1.11[a]
**AST (IU/L)**	47.54±1.53[b]	46.98±1.98[b]	41.29±3.15[ab]	41.13±2.20[ab]	42.91±2.85[ab]	34.73±1.51[a]
**UA (μmol/L)**	243.06±10.02[b]	223.07±5.50[b]	189.15±2.07[a]	213.13±6.04[ab]	227.49±6.24[b]	211.20±4.98[a]
**CRE (μmol/L)**	167.37±9.75[b]	146.00±2.40[ab]	130.65±1.84[a]	123.85±1.31[a]	153.12±3.81[b]	138.08±2.34[a]
**ALB (g/L)**	23.92±2.33[a]	22.70±2.28[a]	22.06±1.25[a]	22.97±1.95[a]	21.45±1.06[a]	15.47±1.28[a]
**T-BIL (μmol/L)**	2.67±0.77[a]	4.50±1.30[a]	1.56±0.45[a]	1.92±0.55[a]	1.63±0.47[a]	2.10±0.560[a]

AST, aspartate aminotransferase; ALT, alanine aminotransferase; UA, uric acid; CREA, creatinine; ALB, albumin; T-BIL, total bilirubin. Data represent mean ± SE (n = 6 to 8 mice/group). Data were analyzed using one-way ANOVA analysis with Tukey's post hoc test or the non-parametric Kruskal-Wallis test with Dunnett's T3 multiple comparison test. Superscript characters indicate significant variation between different values within a row. CHOW: normal diet + sterile water; HFD: high-fat diet + sterile water; CMP: high-fat diet + 100 mg/kg CMP; SeCMP-50: high-fat diet + 50 mg/kg SeCMP; SeCMP-100: high-fat diet + 100 mg/kg SeCMP; SeCMP-200: high-fat diet + 200 mg/kg SeCMP.

Table S3. Group significance using ANOSIM analysis based on Bray-Curtis distance.

**Group 1**	**Group 2**	**Sample size**	**Permutations**	**R***	***p*-value**	***q*-value#**
CHOW	HFD	11	999	0.970667	0.002	0.006
CHOW	CMP	11	999	0.989333	0.002	0.006
CHOW	SeCM-50	11	999	1	0.006	0.015
CHOW	SeCM-100	11	999	0.973333	0.002	0.006
CHOW	SeCM-200	11	999	1	0.002	0.006
HFD	CMP	12	999	0.32963	0.015	0.028125
HFD	SeCM-50	12	999	0.582407	0.002	0.006
HFD	SeCM-100	12	999	0.199074	0.055	0.091667
HFD	SeCM-200	12	999	0.298148	0.015	0.028125
SeCM-50	CMP	12	999	0.051852	0.277	0.377727
SeCM-50	SeCM-100	12	999	-0.01019	0.563	0.603214
SeCM-50	SeCM-200	12	999	0.096296	0.194	0.291
SeCM-100	CMP	12	999	-0.02593	0.465	0.536538
SeCM-100	SeCM-200	12	999	-0.11111	0.803	0.803
SeCM-200	CMP	12	999	0.031481	0.307	0.38375

* R ranges $-1 \leq R \leq 1$. The more R approaches −1, the worse grouping; the more R approaches 1, the better grouping. # Q-value represents the adjust *p* value. CHOW: normal diet + sterile water; HFD: high-fat diet + sterile water; CMP: high-fat diet + 100 mg/kg selenium-deficient *C. militaris* crude polysaccharides; SeCMP-50: high-fat diet + 50 mg/kg SeCMP; SeCMP-100: high-fat diet + 100 mg/kg SeCMP; SeCMP-200: high-fat diet + 200 mg/kg SeCMP.

Table S4. Group significance by PERMANOVA analysis based on Bray-Curtis distance.

Group 1	Group 2	Sample size	Permutations	pseudo-F*	*p*-value	*q*-value#
CHOW	HFD	11	999	4.419134	0.004	0.01
CHOW	CMP	11	999	3.864	0.003	0.01
CHOW	SeCM-50	11	999	5.791691	0.004	0.01
CHOW	SeCM-100	11	999	3.896248	0.004	0.01
CHOW	SeCM-200	11	999	3.992201	0.002	0.01
HFD	CMP	12	999	2.22121	0.013	0.027857
HFD	SeCM-50	12	999	3.503364	0.002	0.01
HFD	SeCM-100	12	999	1.674476	0.067	0.111667
HFD	SeCM-200	12	999	2.210875	0.018	0.03375
SeCM-50	CMP	12	999	1.232954	0.252	0.343636
SeCM-50	SeCM-100	12	999	0.791944	0.742	0.795
SeCM-50	SeCM-200	12	999	1.321897	0.174	0.261
SeCM-100	CMP	12	999	0.851569	0.553	0.638077
SeCM-100	SeCM-200	12	999	0.631252	0.816	0.816
SeCM-200	CMP	12	999	0.988005	0.42	0.525

* pseudo-F the higher, the better grouping. # Q-value represents the adjust *p* value. CHOW: normal diet + sterile water; HFD: high-fat diet + sterile water; CMP: high-fat diet + 100 mg/kg selenium-deficient C. militaris crude polysaccharides; SeCMP-50: high-fat diet + 50 mg/kg SeCMP; SeCMP-100: high-fat diet + 100 mg/kg SeCMP; SeCMP-200: high-fat diet + 200 mg/kg SeCMP.

Table S5. Q-RCP primer sequences.

Target	Direction	Primer sequence (5'-3')
GAPDH	Forward	GCATCCACTGGTGCTGCC
	Reverse	TCATCATACTTGGCAGGTTTC
FAS	Forward	CAAGTGCAAACCAGACTTCTAC
	Reverse	GCACTTTCTTTTCCGGTACTTT
ACC-1	Forward	CCCAGAGATGTTTCGGCAGTCAC
	Reverse	GTCAGGATGTCGGAAGGCAAAGG
SREBP1c	Forward	TCCACCATCGGCACCCACTG
	Reverse	GGCACTGGCTCCTCTTTGATTCC
MTP	Forward	TTGTGTGTGATCTCCGTATTCA
	Reverse	GTTGTAAAGACGGTCTCAGGTA
CYP7A1	Forward	GTGATGTTTGAAGCCGGATATC
	Reverse	TTTATGTGCGGTCTTGAACAAG
CPT1	Forward	CTACATCACCCCAACCCATATT
	Reverse	GATCCCAGAAGACGAATAGGTT
HSL	Forward	GCTGGGCTGTCAAGCACTGT
	Reverse	GTAACTGGGTAGGCTGCCAT
ATGL	Forward	CAGAGATGGACTTCGATTCCTT
	Reverse	CAGGTGCTCTAGAATTCGATCT
FATP	Forward	CCTCTCTGTTCTGATTCGTGTT
	Reverse	GTCCAGCATATACCACTACTGG
TNF-α	Forward	TAGCCAGGAGGGAGAACAGA
	Reverse	TTTTCTGGAGGGAGATGTGG
IL-10	Forward	TTCTTTCAAACAAAGGACCAGC
	Reverse	GCAACCCAAGTAACCCTTAAAG
IL-6	Forward	CTCCCAACAGACCTGTCTATAC
	Reverse	CCATTGCACAACTCTTTTCTCA
ZO-1	Forward	CTGGTGAAGTCTCGGAAAAATG
	Reverse	CATCTCTTGCTGCCAAACTATC
Ocln	Forward	TGCTTCATCGCTTCCTTAGTAA
	Reverse	GGGTTCACTCCCATTATGTACA
